# Impact of Periodontitis on Glycemic Control and Metabolic Status in Diabetes Patients: Current Knowledge on Early Disease Markers and Therapeutic Perspectives

**DOI:** 10.1155/2022/4955277

**Published:** 2022-08-13

**Authors:** Simona Santonocito, Alessandro Polizzi, Enrico Marchetti, Domenico Dalessandri, Marco Migliorati, Saturnino Marco Lupi, Marco Cicciù, Gaetano Isola

**Affiliations:** ^1^Department of General Surgery and Surgical-Medical Specialties, School of Dentistry, University of Catania, Via S. Sofia 78, 95124 Catania, Italy; ^2^Department of Life, Health and Environmental Sciences, University of L'Aquila, L'Aquila, Italy; ^3^Department of Medical and Surgical Specialties, Radiological Sciences and Public Health, University of Brescia, Brescia, Italy; ^4^Dental School, Department of Surgery, University of Genova, Italy; ^5^Unit of Oral Surgery and Implantology, Section of Dentistry, Department of Clinical, Surgical, Diagnostic and Pediatric Sciences, University of Pavia, 27100 Pavia, Italy; ^6^Department of Biomedical and Dental Sciences, Morphological and Functional Images, University of Messina, Messina, Italy

## Abstract

Diabetes mellitus and periodontitis are two of the most common chronic diseases affecting the world's population, and they are intimately linked. For several years, in fact, it has been known that there is an interdependent relationship between the two diseases: Diabetes promotes the destruction of periodontal tissues, and periodontal disease negatively affects glycemic control. In relation to the control of dental plaque and oral dysbiosis responsible for periodontal disease, both nonsurgical and surgical therapy associated with proper home hygiene procedures have emerged as essential for good glycemic control. Moreover, several evidences suggest the essential role played by the control of periodontal disease in preventing the onset of the most common complications of diabetes: cardiovascular diseases, retinopathies, and other systemic diseases. The aim of this study is to update the current knowledge on the bi-univocal relationship between diabetes and periodontitis and the impact of therapy in the optimal management of these two disorders. From the information found in the literature, it has emerged that the correct treatment of periodontal disease in diabetic patients represents one of the main mechanisms and means currently established and valid to control periodontal disease and glucose metabolism and prevent the onset or development of diabetic complications.

## 1. Introduction

Diabetes mellitus is a metabolic disease characterized by serum hyperglycemia due to insulin deficiency, insulin action or both [1]. The International Diabetes Federation reported a global diabetes prevalence in 2019 estimated about 463 million patients aged between 20 and 79 years and that this prevalence was triplicated in almost 20 years [2]. There are two main categories of diabetes. Type 1 diabetes is characterized by autoimmune destruction of pancreatic *β* cells inducing insulin deficiency and hyperglycemia. This form of diabetes is typical of children and adolescents and represent about 5-10% of the total cases of diabetes [3]. Type 2 diabetes, representing about 90-95% of the total cases of diabetes, is typical of adults, and it is characterized by both insulin deficiency and insulin resistance [4]. Genetic factors and environmental triggers (such as enteroviruses) are the causes of type 1 diabetes, whereas type 2 diabetes is correlated to subjects' lifestyles, physical activity, and diet [3].

Diabetes and serum hyperglycemia lead to different systemic complications impacting the quality of life. More specifically, it is known that diabetic patients may suffer from various disorders such as cardiovascular disease, neuropathies, nephropathies, retinopathies, cerebrovascular diseases, macro- and microvascular diseases, and impaired immune function with incremented risk of infections [3]. In this regard, since the 1990s, periodontitis has been considered another classical complication of diabetes [5].

Periodontitis is a chronic inflammatory disease that affects periodontal tissues causing attachment loss, exposure of the tooth root, and tooth loss. It is the result of the interaction between a dysbiotic oral microbiota and a host with an impaired immune response [6–8]. In particular, the inflammatory destruction of periodontal tissues is characterized by impaired release of different inflammatory mediators such as interleukins IL-1*β* and IL-6, tumor necrosis factor alpha (TNF-*α*), prostaglandin E2 (PGE2), metalloproteinases, adipokines, and chemokines [9, 10]. Furthermore, type 1 and type 2 diabetes are characterized by higher systemic inflammatory state contributing to micro- and macroangiopathies and increased oxidative stress. Higher levels of IL-6, TNF-*α,* and C reactive protein (CRP) have been found in diabetic patients, and it is known that high CRP levels are correlated to insulin resistance due to impaired intracellular insulin signaling. Moreover, higher CRP and IL-6 levels may be found in patients affected by periodontitis and an association between IL-6 levels, and the extent of periodontitis has been demonstrated. Therefore, some recent studies have shown that a systemic inflammatory state related to periodontitis could increase the risk to develop diabetes [10, 11]. To date, the scientific community commonly accepts the idea of a two-way relationship between diabetes and periodontitis. Epidemiological and animal studies have shown that the presence of one disease increases the risk and severity of the other [5, 12].

Diabetes treatment aims to control serum glucose levels and its related complication. The most effective therapeutic strategy is the one that also involves the direct collaboration of the patient through a correct lifestyle and healthy eating and physical activity habits. Similarly, periodontal treatment aims to reestablish eubiosis of the oral microbiota and control of the subgingival biofilm, reducing the progression of the disease. Also in this case, the best therapeutic approach is the one that actively involves the patient through proper home oral hygiene and a healthy lifestyle [3].

In this regard, the aim of this review is to update the current knowledge on the two-way relationship between diabetes and periodontitis and on the impact of therapy in the optimal management of these two disorders.

## 2. Diabetes and Its Impact on Periodontal Health

Epidemiologic studies suggested an increased risk of periodontal diseases in diabetic patients [4]. It has been estimated that diabetic individuals have 2-3 times incremented risk to develop periodontal disease [13]. The key factor increasing this risk is the quality of glycemic control. More specifically, the US National Health and Nutrition Examination Survey (NHANES) III revealed that people with glycated hemoglobin (HbA1c) >9% showed a significant greater prevalence of sever periodontal disease compared to individuals without diabetes after adjusting for confounding factors [14]. An important contribution in defining the prevalence of diabetes in patients with periodontal disease is due to studies conducted first by Cianciola et al. and later by Lalla et al. [15, 16]. In detail, these studies examined the periodontal status of a large cohort of children and adolescents with diabetes, aged 6-18 years. The results showed that subjects with diabetes had greater gingival inflammation and attachment loss than healthy controls, concluding that there is an association between diabetes and an increased risk of periodontal destruction even very early in life [15, 16].

In addition, since the 1990s, numerous cross-sectional and longitudinal studies indicated that diabetes is a risk factor for developing periodontal disease [4, 11]. For example, a two-year follow-up study individuated 2.6 incremented risk of periodontal disease in diabetic Pima Indians of Arizona compared to nondiabetic subjects [17]. Curiously, a study evaluating the impact of hypoglycemic therapy on periodontal tissues revealed that the reduction of HbA1c levels over the six months period improved bleeding on probing (BOP) without periodontal therapy [18]. Furthermore, numerous studies showed that poor glycemic control is associated to a greater risk of developing severe periodontitis compared to well-controlled subjects with diabetes [19–23].

Most of the studies have been carried out on patients with type 2 diabetes mellitus. However, type 1 diabetes also increases the risk of periodontitis in children and adolescents as well [11]. For example it has been reported that about 10% of type 1 diabetic children had incremented bone and attachment loss compared to healthy controls, in spite of similar plaque score among the groups [15]. Furthermore, a study comparing 350 diabetic vs 350 nondiabetic children reported an increased number of pathological periodontal sites in diabetic subjects compared to normal controls (>20% vs 8% of sites) [16].

**Figure 1:**
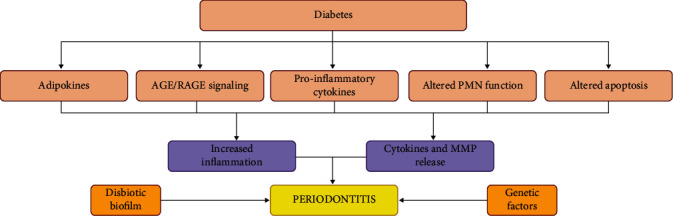
Impact of diabetes on periodontal health. Hyperglycemia in diabetic subjects favors different pro-inflammatory responses that affect the systemic health and the periodontal tissues. The incremented adipokine production (especially in obese adults) induces the release of different pro-inflammatory mediators such as leptin, IL-6, and TNF-*α*. Furthermore, AGEs deposition in the different human tissues further stimulates cytokine release and inflammation. Another mechanism of periodontal tissue destruction is the altered neutrophil polymorphonucleates' (PMN) function characterized by enhanced respiratory burst and delayed apoptosis. MMP is the acronym of metalloproteinases. All these factors combined to genetic predisposition and pathological subgingival biofilm lead to the increased risk of severe periodontitis development.

Diabetes increases the phlogistic process in the periodontal tissues though different mechanisms. For example, in diabetic patients, the incremented production of advanced glycation end-products (AGEs) lead to their deposition and interaction with their receptors (RAGEs) in the periodontal tissues, activating the local inflammation. More specifically, AGEs are irreversible non-enzymatically glycated and oxidated proteins, and lipids accumulated in the serum and tissues of diabetic subjects. The AGEs/RAGEs interaction increase the activity of monocytes/macrophages and endothelial cells, inducing the release of pro-inflammatory cytokines such as interleukins (IL)-1*β*, IL-6, and tumor necrosis factor (TNF)-*α* [24, 25]. The induced release of cytokines and disruption of the receptor activator of NF-*κ*B ligand/osteoprotegerin (RANKL/OPG) axis increase bone resorption [10, 26]. This exacerbated inflammation, associated to subgingival biofilm formation, increases the periodontal inflammation and attachment and bone loss [27, 28]. Conversely, animal studies revealed that blocking RAGEs induced the suppression of alveolar bone loss in diabetic mice with induced periodontal disease [29]. Furthermore, it has been shown that subjects with periodontal disease and diabetes had increased levels of RAGEs compared to nondiabetic patients with periodontal disease [30].  Figure 1 resumes the pathogenetic mechanisms though which diabetes may have an impact on periodontal health. It is well known that advanced periodontal disease is characterized by a vascular response induced, in part, by the deposition of AGEs at the level of the basement membranes, with an increase in their thickness, which would hinder not only the exchange of nutrients in the periodontal tissues, but also the arrival of immune cells (PMNs) at the periodontal epithelia with the bacterial biofilm [31]. Thus, the increased susceptibility of diabetic patients to develop periodontal disease would be linked to disturbances in neutrophil function, elevated blood glucose levels, disturbances in collagen synthesis, maturation and degradation, and gingival microangiopathy. Diabetes-associated angiopathy may result in the proliferation of blood vessels and increased deposition of advanced glycosylation end products (AGEs) in the basement membrane [31–33].

## 3. Impact of Periodontitis on Metabolic Status during Diabetes

**Figure 2:**
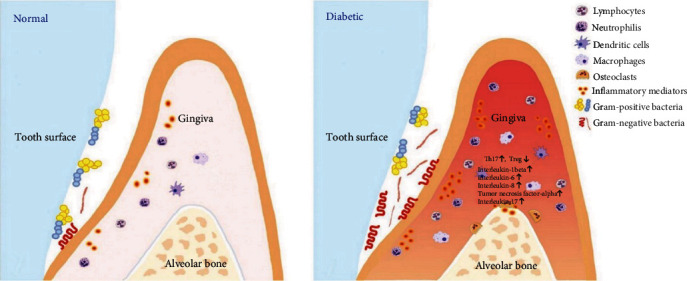
Diabetes enhances inflammation in the periodontium leading to changes in bacterial composition. Diabetes results in more inflammation mirrored by increased leukocytes and cytokine expression, as well as a change in bacterial composition that improves the overall pathogenicity of the microbiota [42].

The bidirectional link between diabetes and periodontitis is now well recognized: Di!!abetes is a predisposing factor for periodontal disease, and periodontal disease affects metabolic control in patients with diabetes, increasing the risk of development complications, like cardiovascular diseases and retinopathy and kidney diseases [34]. According to the most accredited theory, the expression and passage into the systemic circulation, from periodontal tissue, of the pro-inflammatory cytokines typical of periodontal disease, are at the root of the exacerbation and worsening of diabetes mellitus [35]. The National Health and Nutrition Examination Survey (NHANES) III showed that there is a linear relationship between the destruction of periodontal disease and the severity of insulin resistance [36]. One of the first studies to investigate the effect of periodontitis on glycemic control was conducted on the Indian Gila River population, which has a high prevalence of type 2 diabetes. The study found that patients with diabetes and severe periodontal disease had poorer glycemic control after 2 years of follow-up than patients without or with mild periodontal disease [37]. A further study, also conducted in the Gila River population, observed that death from cardiovascular disease and diabetic nephropathy was higher in individuals with diabetes and periodontitis than in those with diabetes and little or no periodontitis, indicating that periodontitis was a predictor of future ischemic heart disease and diabetic nephropathy, again in relation to parameters for age, sex, duration of diabetes, glycated hemoglobin, macroeconomic study, body mass index, cholesterol, hypertension, electrocardiogram abnormalities, and smoking [38]. The high vascularity of inflamed periodontal tissue may liken it to an endocrine-like source for TNF-*α* and other mediators of inflammation, with the ulcerated epithelium of the periodontal pocket representing a chronic source of systemic challenge from bacteria, bacterial products, and locally produced inflammatory mediators [39, 40]. TNF-*α*, IL6, and IL1 are the main inflammatory mediators of periodontal disease, which adversely affect glucose and lipid metabolism, particularly following an acute infectious challenge or trauma [41] (Figure 2).

**Figure 3:**
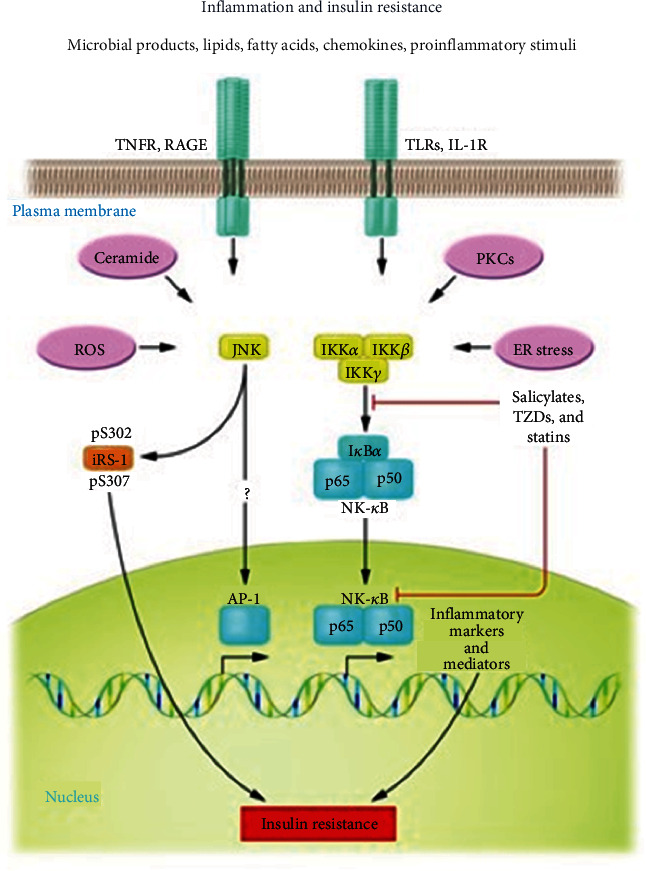
An explanatory graph of the different molecular pathways referring mainly to the blockade of the insulin receptor ISR-1 by inflammatory mediators (TNF-alpha).

The molecular basis of altered insulin action secondary to increased inflammatory signaling involves the inhibition of signaling pathways downstream of the insulin receptor, in which the c-JUN N-terminal Kinase (JNK1) axis and the inhibitor of nuclear factor kappa-B kinase (IKK*β*)/NF*κ*B are involved. Inflammatory mediators including some interleukins and PCR, induce stimulation of JNK and MAPK, resulting in reduced phosphorylation of IRS-1 substrate tyrosine sites with consequent inhibition of PI3 and Akt enzyme activation. This results in the failure to translocate glucose transporter type 4 (GLUT4) across the plasma membrane, preventing insulin signal transduction. Another mechanism by which insulin resistance can occur involves another inflammatory kinase IKK-*β*, which through direct serine phosphorylation of IRS-1 and phosphorylation of the NF-*Κβ* inhibitor induces the production of several inflammatory mediators, including TNF*α* and IL-6, which inhibits serine phosphorylation of IRS-1 and thus the translocation of the glucose transporter (GLUT 4) [35, 43]. TNF-*α* has been shown to affect lipid metabolism and is an insulin antagonist [44]. The increased degradation of free fatty acids in diabetics also promotes the development of systemic inflammation-mediated insulin resistance. Ectopic fat loss activates PCK which inhibits the activation of IRS1 serine kinase, preventing the translocation of glucose transporters. Furthermore, the systemic pro-inflammatory environment also induced by the presence of TNF-*α* determines a reduced release of adiponectin into the cells. Adiponectin is a molecule produced by adipocytes with potent anti-inflammatory activity and sensitizes cells to insulin. So, it also inhibits adiponectin production in adipocyte cells. Adiponectin is a molecule produced by fat cells that has anti-inflammatory effects, by antagonizing the action of TNF, and sensitizing the cells to insulin. However, the pathways involved in this mechanism still need to be further investigated. Probably, PPAR-*α* and other signal transduction pathways are not yet known and play a role in enhancing insulin sensitization [35] (Figure 3). In conclusion, the systemic inflammation caused by periodontal disease triggers the activation of several pathways capable of altering glucose metabolism, favoring the formation of advanced glycation end-products that, by binding to their receptors, induce further release of inflammatory mediators, oxidative stress, and apoptosis, creating a true vicious circle. It is therefore the systemic inflammatory response that negatively influences glycemic control and promotes the development of complications in periodontitis patients [45].

## 4. Therapeutic Perspectives

Over the past few years, several RTC studies have been performed to investigate the response of diabetic subjects with periodontal disease to periodontal treatment. In one RTC study, the impact of nonsurgical periodontal therapy in patients with poorly controlled diabetes and severe periodontitis was evaluated. Patients were divided into 5 groups, each of which underwent 5 different methods of periodontal therapy, including systemic minocycline and topical antimicrobial agents, as well as scaling and root planing under local anesthesia and hopeless tooth extraction. The study showed that glycated hemoglobin levels decreased in the groups that received systemic antibiotics and topical chlorhexidine, extraction and scaling, and root planing treatment, compared to those that received only extensive scaling and root planing. The results of this study indicated that proper management of periodontal disease allows for more effective management and control of uncontrolled diabetes by reducing hyperglycemia [46]. Further studies have confirmed and improved on these results and have also shown that periodontal therapy has an impact on reducing glycated hemoglobin. In fact, several studies have observed a reduction in the glycated hemoglobin value 3-4 months after nonsurgical periodontal treatment, ranging from 0.27% to − 1.03%. At 6 months after periodontal treatment, the reduction in glycated hemoglobin was reduced to values between 0.02% and − 1.18% [47, 48]. There was also a reduction in fasting plasma glucose, ranging from − 8.95 to − 9.04 mg/dL, at 3-4 months. This reduction in hyperglycemia, if prolonged, could reduce diabetic complications and improve quality of life [47, 48]. At this time, there is no clear evidence that periodontal treatment combined with local or systemic antibiotic therapy is more effective in inducing better glycemic control in diabetic and periodontal patients. In fact, the many studies carried out have not reached a unanimous evidence, as shown by a recent review [39]. Other studies evaluated a possible collaboration between the efficacy of brushing on plaque levels and glycated hemoglobin, observing that subjects with inadequate brushing had higher levels of plaque and glycated hemoglobin than subjects with greater brushing efficiency. These studies show that home oral hygiene maneuvers and nonsurgical periodontal therapy sessions are valuable practices in controlling the levels of glycated hemoglobin and, consecutively, to be considered as preventive maneuvers to prevent the onset of complications typical of diabetes [49]. Recently, a very interesting study evaluating the role of local inflammation in systemic inflammatory burden and glycemic control in people with type 2 diabetes indicated an improvement in periodontal health (reduction in pocket depth and bleeding on probing and increase in attachment level) and blood glucose levels (fasting serum glucose and glycated hemoglobin), after a combination of nonsurgical and surgical treatment with additional use of systemic and local antibiotics, hopeless tooth extraction, and treatment of acute dental disease (e.g., caries and endodontic lesions). The duration of the study was 12 months, and the patients were evaluated bimonthly [50]. We can therefore see that the improvement in glycemic levels observed following periodontal treatment represents evidence that the infection and inflammation associated with periodontal disease significantly affect the inflammatory picture [51].

## 5. Conclusions

In accordance with the results of the analysis of the current literature on the subject, inflammation is the common pathway linking diabetes to periodontal disease. In fact, the reduction of periodontal inflammation, induced by both surgical and non-surgical periodontal therapy, makes it possible to better control serum glucose and glycated hemoglobin levels, thus reducing the risk, which is significantly increased in periodontal and diabetic patients, of developing kidney, heart, etc. complications. At the same time, better control of glycated hemoglobin and serum glucose reduces the extent of periodontal destruction. Therefore, the control of inflammation is the cornerstone for managing diabetes-periodontitis risk due to the increased production of advanced glycation end-products (AGEs) and interaction with their receptors (RAGEs) in periodontal tissues, which is responsible for the increased activity of monocytes/macrophages, endothelial cells, the release of pro-inflammatory cytokines, and, finally, the activation of NF-*κ*B receptor ligand/osteoprotegerin (RANKL/OPG). All this induces increased bone resorption. This, when associated with the presence of a subgingival biofilm, increases periodontal inflammation. In conclusion, we can state that the correct treatment of periodontal disease in diabetic patients represents one of the main mechanisms and means currently established and valid for controlling periodontal disease and glucose metabolism and preventing the onset or development of diabetic complications.

## Data Availability

Data are available from the corresponding author upon reasonable request.
